# Seroprevalence of major respiratory diseases of chickens in central Ethiopia in different chicken production systems

**DOI:** 10.1016/j.psj.2022.102065

**Published:** 2022-07-26

**Authors:** Tadiose Habte, Priscilla F. Gerber, Fozia Ibrahim, Peter J. Groves, Stephen W. Walkden-Brown

**Affiliations:** ⁎ National Poultry Research Program, Ethiopian Institute of Agricultural Research, Debrezeite, Ethiopia; † Animal Science, School of Environmental and Rural Science, University of New England, Armidale, NSW, Australia; ‡ Armauer Hansen Research Institute, Addis Ababa, Ethiopia; § Sydney School of Veterinary Science, Poultry Research Foundation, Faculty of Science, the University of Sydney, Australia

**Keywords:** seroprevalence, respiratory pathogens, fengil, Ethiopia

## Abstract

In Ethiopia, most chicken disease outbreaks and mortalities are attributed to a respiratory syndrome known as “fengil” with variable clinical signs and undefined etiology. The main goal of this study was to determine whether key respiratory pathogens that could contribute to the fengil syndrome circulate in Ethiopia. Specifically, we aimed to determine the seroprevalence of infectious laryngotracheitis virus (**ILTV**), infectious bronchitis virus (**IBV**), Newcastle disease virus (**NDV**), *Mycoplasma gallisepticum* (**Mg**), and avian metapneumovirus (**aMPV**). A cross-sectional survey was conducted in 158 scavenging and 42 small and medium-scale intensive chicken holdings in the East, West and North Shewa Zones of central Ethiopia. Blood from 495 chickens was collected and serological tests were used to determine exposure to these pathogens. Vaccination against NDV was the only immunization practiced with a significantly higher vaccination rate in the intensive than the scavenging system. Serological evidence of a high level of exposure to all pathogens was detected, including the first report on the seroprevalence of aMPV, ILTV, and IBV in the East Shewa Zone. The chicken and holding seroprevalence rates were respectively 91% and 94% for IBV, 34% and 57% for aMPV, 47% and 66% for Mg, 27% and 51% for ILTV and in unvaccinated flocks, 39% and 53% for NDV. These pathogens could contribute to the fengil syndrome, commonly ascribed to NDV. The seroprevalence of aMPV and ILTV was higher in chickens under the scavenging system. Exposure to multiple pathogens was common, with more than 50% of chickens positive for three or more pathogens in the scavenging system. This was reflected in significant positive associations between seropositivity to ILTV, Mg, ILTV, and IBV. The role of these pathogens in the causation of respiratory disease in the field requires further investigation.

## INTRODUCTION

In Ethiopia, poultry are the second most economically important livestock after cattle (Dessie and Ogle, 2001). With decreasing grazing land available per capita, poultry production is considered one of the principal means of providing the poor and small landholders with good quality animal protein (Shapiro et al., 2015). The scavenging chicken production system which is the predominant production system in Ethiopia is characterized by small flock size, minimal inputs (housing feed and health care) and very low output (Dessie, 1997). The breeds involved in the scavenging production system are predominantly local breeds and crosses between local and exotic breeds, with less than 10% of the chickens being of imported breeds (Habte et al., 2015). Intensive fully housed production systems are also common in Ethiopia, with small-scale intensive production widely practiced in urban and periurban areas (Alemu et al., 2008; Kryger et al., 2010). The breeds used in this system tend to be improved commercial laying, meat, and dual-purpose breeds. The improved breeds frequently do not perform to their genetic potential due to suboptimal nutrition and disease prevalence in the commercial system (Habte et al., 2017).

Disease-related mortality among chickens in Ethiopia is high and a major constraint (CSA, 2021). A participatory rural appraisal study by Sambo et al. (2015) identified feed shortage, disease, lack of improved breeds, poor markets, poor veterinary services, inadequate shelter, low productivity, and predators as significant constraints for the industry. Among the constraints assessed disease was identified as the most important production constraint in the scavenging system and the second most important in the intensive system. Feed shortage was identified as the most important constraint in the intensive system and second in the scavenging production system. The village production system significantly increases the risk of transmission of diseases since there is a high chance of contact with wild birds, other chickens and disease vectors with little or no biosecurity or control over the ranging area. The vaccination service for village chickens in Ethiopia is irregular and is mainly a government extension service, in which only a live Newcastle disease vaccine is provided in some parts of the country (Asfaw et al., 2021). Vaccination is more widely adopted in the intensive production system, typically for NDV, Gumboro disease (infectious bursal disease), and fowl typhoid (*Salmonella gallinarum*).

Globally, respiratory diseases are arguably the most important group of diseases in chickens, causing high mortality and production loss in different production systems (Hafez, 2002; Shankar, 2008). Multiple etiology and complexity are characteristic features of respiratory disease in poultry (Malik et al., 2004; Roussan et al., 2008; Mazengia, 2012). Among the major pathogens affecting the respiratory tract of chickens worldwide is Newcastle disease virus (**NDV**), infectious laryngotracheitis virus (**ILTV**), infectious bronchitis virus (**IBV**), *Mycoplasma gallisepticum* (**Mg**), *Mycoplasma synoviae* (**Ms)**, avian metapneumovirus (**aMPV**), avian influenza virus, *Pasteurella multocida, Aspergillus fumigatus*, and *Avibacterium paragallinarum* (Brown Jordan et al., 2018; Bhuiyan et al., 2019; Batista et al., 2020; Swayne, 2020).

Understanding the epidemiology of each disease is vital for developing and evaluating control measures. In the traditional Ethiopian village production system, a disease with pneumotropic Newcastle disease-like symptoms is referred to as “fengil” respiratory disease. Disease-related losses are commonly attributed to this condition. There were more than 40 “fengil” outbreaks in 2017 in central Ethiopia, but the causative agent for those cases has not been ascertained, and other pathogens may be involved. Studies on respiratory pathogens in Ethiopia are scant, and most have focused on single causative agents, whereas concurrent infection with multiple agents could occur (Gharaibeh and Algharaibeh, 2007; Haji-Abdolvahab et al., 2019).

Previous studies in Ethiopia have shown that NDV has a seroprevalence between 5.9 and 30% in village chickens that were presumably unvaccinated (Nasser et al., 2000; Tadesse et al., 2005; Zeleke et al., 2005; Chaka et al., 2012; Mazengia, 2012; Chaka et al., 2013; Sori et al., 2016; Mamo and Yimer 2021). Serological evidence of IBV and aMPV subtype B has been reported in imported chicken breeds at Debre Zeit Agricultural Research Centre (Hutton et al., 2017), but the occurrence of those pathogens outside this research facility is not known. The same study reported the detection of Mg and IBV nucleic acids from oropharyngeal swabs in the same research flock (Hutton et al., 2017). Previous seroprevalence studies have reported seroprevalence of 24% for IBV in scavenging and intensive chickens of north-west Ethiopia (Birhan et al., 2021), 65% for ILTV in East Shewa and South Shewa (Tesfaye et al., 2019; Roba et al., 2020), and 66% for Mg in East Shewa (Jibril et al., 2018). There has been ongoing active surveillance for highly pathogenic avian influenza (**HPAI**) in Ethiopia since 2008, and it has never been reported in the country (Ali, 2014; Etsegenet Abera, Ministry of Agriculture, 2021). Since there is no active surveillance for respiratory pathogens except HPAI and limited studies on their prevalence and distribution, it is important to monitor their status in the country.

This study aimed to ascertain the prevalence of respiratory pathogens in poultry-producing regions around the capital of Ethiopia and their possible contribution to the fengil syndrome. The overall proposition under test was that non-Newcastle disease respiratory infections are prevalent in chicken flocks of all production systems, potentially contributing to disease outbreaks identified as fengil. Specific subsidiary propositions were 1) Serological evidence of infection with aMPV, ILTV, IB, Mg and NDV will be found to be widespread in the surveyed region; 2) The seroprevalence of aMPV, IBV, ILTV, Mg, and NDV will differ between the production systems and breeds evaluated; 3) Serological evidence of exposure to multiple agents will be common, and the exposure to ILTV, IBV, NDV, Mg, or aMPV will predispose to infection with each other and 4) Seroprevalence of NDV infection will be higher in vaccinated than unvaccinated flocks.

## METHODS

### Ethical Approval and Study Design

The research protocols used in this study were approved by the University of New England Human Ethics Committee (approval number HE19-208) and the Animal Ethics Committee (approval number AEC19-047).

### Study Area and Selection Strategy

A cross-sectional serological survey was conducted in scavenging, and small to medium-scale intensive chicken production systems in 3 zones (East Shewa, West Shewa, and North Shewa) of central Ethiopia. Holdings with less than 50 chickens that scavenge with or without night shelter were considered scavenging. Holdings with more than 50 chickens, fed commercial feed and fully housed were considered intensive. In Ethiopia, the largest administrative units are regions, then zones and then Woreda (districts). The Central Ethiopia region includes the capital city Addis Ababa and the East Shewa, West Shewa, and North Shewa zones. These 3 zones are located within a 100 km radius from the center of Addis Ababa, with altitudes ranging from 1,700 meters in East Shewa to 2,850 meters in North Shewa. The average annual temperature in the selected zones lies between 15 and 19°C. The survey was conducted between July 2019 and April 2020.

These zones were selected for the survey as they have a high chicken population, including intensive production systems, and were accessible from the base laboratory. Districts within the zone were selected based on a significant level of poultry production, the presence of both scavenging and intensive production systems and accessibility. Accessibility was set as a criterion to limit sample transportation times to the laboratory. Villages and households within a district were selected randomly using a lottery system.

A targeted sample size of 302 chickens was determined using an open-source sample size calculator (OpenEpi, Version 3), based on an expected prevalence of 27% based on previous reports for NDV (Chaka et al., 2013), and a 5% level of significance. The actual number of chickens sampled was 520, but 25 serum samples from 7 scavenging (14 sera) and 4 intensive (11 sera) holdings were not subjected to analysis due to poor quality. Therefore, 495 serum samples were used, with their distribution within zones and production systems shown in Table 1.

**Table 1:** Sample size and structure for the serological survey.

Zone	Scavenging system		Intensive system		Total holdings	Total chickens
	Holdings	Chickens	Holdings	Chickens		
East Shewa	55	104	22	134	77	238
North Shewa	72	137	16	44	88	181
West Shewa	30	60	5	16	35	76
Total	157	301	43	194	200	495

The study sampled adult chickens from the scavenging and small-scale intensive production systems. For both production systems, the criteria for inclusion were the willingness to participate and to allow the collection of 1.5 mL of blood from 2 chickens per holding in scavenging systems and 5 chickens per holding in intensive systems. If the holding had both male and female chickens, samples were taken from both. Information such as flock structure, breed, and season of the year was also recorded. In the scavenging production system, family holdings were selected from a local administration list using fixed number random interval selection. If the identified household was not raising chickens or had less than 2 adult chickens at the time of the survey, then the next house on the right of the preselected household was invited to participate in the survey. The small-scale intensive chicken producers were selected based on their availability based on the list of farmers at district-level government offices.

Blood was collected from the brachial wing vein using a 3 mL syringe with a 23-gauge needle and transported to the laboratory at the Armauer Hansen Research Institute (**AHRI**) on the same day of collection. Blood was allowed to clot in the syringe overnight at room temperature before serum separation. The serum was stored at −20°C until laboratory analysis.

### Serological Analysis

Serum samples were tested using commercially available indirect ELISAs (IDvet, France) for the detection of antibodies against ILTV (IDvet ID Screen ILTV indirect, lot F39), NDV (IDvet ID Screen Newcastle disease conventional vaccines lot F41), IBV (IDvet ID Screen infectious bronchitis indirect lot F86), Mg (IDvet ID Screen Mycoplasma gallisepticum indirect, lot F11) and aMPV (IDvet ID Screen aMPV indirect, lot F54) at AHRI as per the manufacturer's protocol. The optical density (OD) value of tested samples was determined using an EMax plus microplate reader (Molecular Devices, LLC MA), at 450 nm for all assays and results were expressed in titers and categorized as positive if the result was greater than the cut off value. The following cut-off antibody titer values were used to categorize the serological result positive for the specific disease, > 396 for aMPV, ≥ 843 for Mg, ≥ 853 for IBV, ≥ 611 ILTV and ≥ 993 for NDV.

### Data Analysis

Categorical data (positive, negative) for each ELISA assay were analyzed using contingency table analysis using JMP 16.0 software (SAS Institute, Cary, NC). Seroprevalence of aMPV, ILTV, IBV, Mg and in the case of NDV within NDV vaccination class (vaccinated, unvaccinated) was analyzed at both the holding and individual chicken level by the Chi-Square test of independence testing the effects of Zone (East, West, and North Shewa) breed (local or cross and exotics) and production system (scavenging, intensive). Due to confounding of the effects of the breed and production system, the effect of breed was tested only within the scavenging production system. At the holding level, a seropositive result was obtained if at least one sampled chicken was positive. Unless otherwise specified, all prevalence results are at the holding level. The odds ratio of being serological positive for one disease to being seropositive for the other diseases was calculated by fitting a nominal logistic model. A significance level of *P*  ≤  0.05 is used throughout. Descriptive statistics are provided for antibody titer distribution and the number of pathogens for which individual chickens were seropositive.

## RESULTS

### Flock Structure and Vaccination Practices in the Different Production Systems

For the scavenging production system the mean, median, and range in flock size were 8, 2, and 2 to 50, respectively. For the intensive production system the equivalent values were respectively 82, 62, and 31 to 199. NDV was the only pathogen tested for which vaccination was available. There was a higher (*P* < 0.0001) NDV vaccination rate in the intensive system (65% of holdings and 78% of birds) than in the scavenging system (12% of holdings and 11% of birds); therefore, data for NDV are presented separately for vaccinated and unvaccinated chickens.

### Antibody Titers and Their Distribution

**Figure 1:**
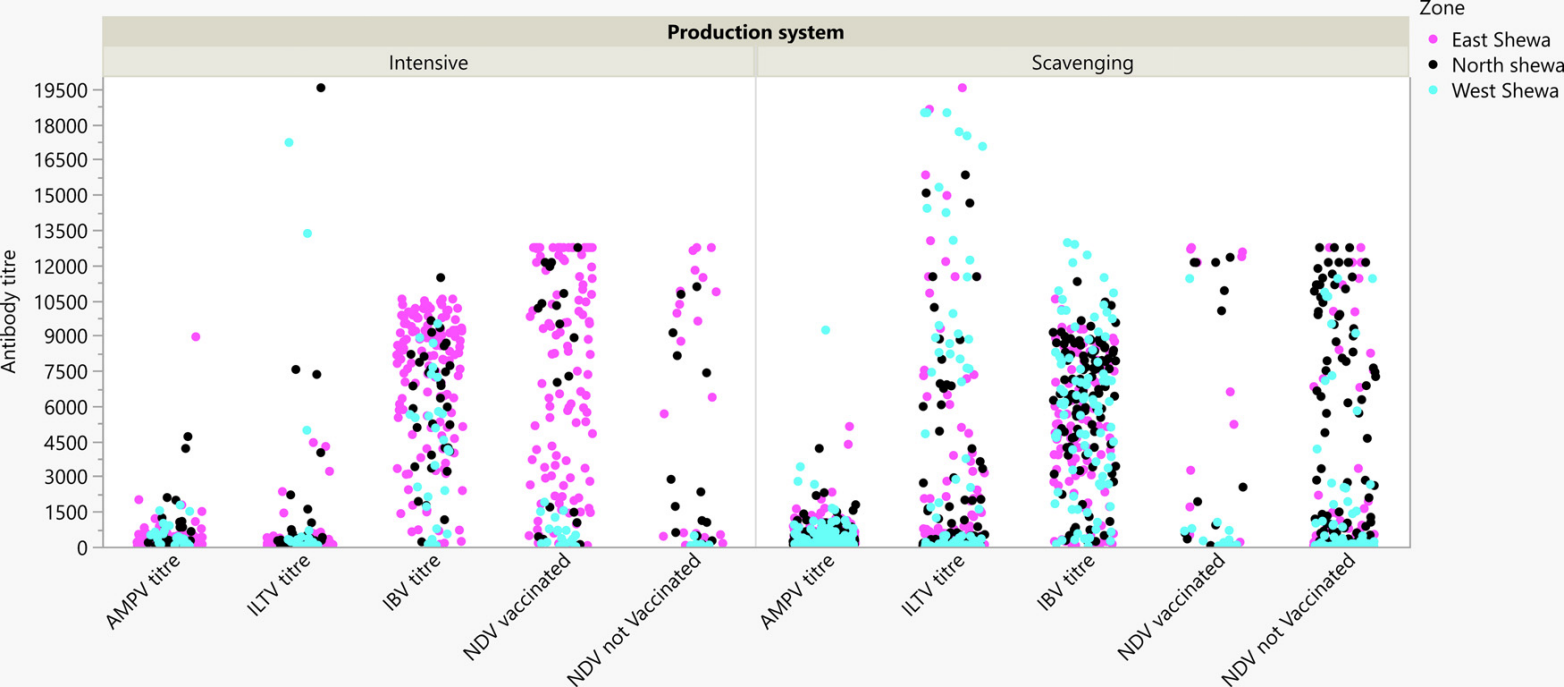
Distribution of individual chicken antibody titer against aMPV, ILTV, IBV, Mg, and NDV (in vaccinated and unvaccinated flocks) in the three zones by the production system. Abbreviations: aMPV, avian metapneumovirus; IBV, infectious bronchitis virus; ILTV, infectious laryngotracheitis virus; Mg, *Mycoplasma gallisepticum*; and NDV, Newcastle disease virus.

The distribution of antibody titers for the five target pathogens within the 2 production systems is shown in Figure 1, and key parameters are shown in Table 2. A wide range of titers was detected for all pathogens, with the data being skewed toward a small number of very high titers, so the median is always lower than the mean (Table 2). Descriptive data are presented with statistical analysis restricted to the prevalence data in the following sections.

**Table 2:** Key statistical parameters and cut-off values for individual chicken antibody titres against aMPV, ILTV, IBV, Mg, and NDV (in vaccinated and unvaccinated flocks) within intensive (I) or scavenging (S) production systems.

	aMPV		ILTV		IBV		Mg		NDV vaccinated		NDV non-vaccinated	
Production system	I	S	I	S	I	S	I	S	I	S	I	S
Mean	378	515	717	2,982	6,677	567	2,169	2,080	6,420	5,000	4,520	2,867
Median	117	278	81	314	7,534	6079	787	875	6,441	1,804	1,109.8	298.6
minimum	0	2	0	0	54	23	4	7	2	48	11	1
maximum	8,941	9,227	21,792	26,588	11,462	12,954	6,722	6,940	12,741	12,741	12,741	12,741
Cut-off value	396		611		853		843		993			

Abbreviations: aMPV, avian metapneumovirus; IBV, infectious bronchitis virus; ILTV, infectious laryngotracheitis virus; Mg, *Mycoplasma gallisepticum*; and NDV, Newcastle disease virus.

### Seroprevalence of the Selected Respiratory Pathogens

The overall seroprevalences by holding of aMPV, ILTV, IBV, and Mg were 56.5, 50.5, 94, and 66%, respectively. Seroprevalence of NDV was 73.9% for NDV vaccinated and 52.6% for unvaccinated holdings (Table 3). The overall seroprevalences in individual chickens were 33.5, 26.9, 90.5, and 47.1% for aMPV, ILTV, IBV, and Mg, respectively. Seroprevalence of NDV was 73% for NDV-vaccinated and 39% for unvaccinated chickens (Table 4). The effects of zone, production system, and breed for each pathogen are described below.

**Table 3:** Holding level seroprevalence of aMPV, ILTV, IBV, Mg, and NDV in three Zones of central Ethiopia.

Zones	Total holdings	aMPV positive		ILTV positive		IBV positive		Mg positive		NDV positive (vaccinated)		NDV positive (non-vaccinated)	
		N	%	N	%	N	%	N	%	N	%	N	%
East Shewa	77	47	61.0	39	50.7	71	92.2	42	54.6^a^	17/21	81.0^x^	21/56	37.5^a^^y^
North Shewa	88	43	48.9	45	51.1	86	97.7	65	73.9^b^	13/19	68.4	46/67	68.7^b^
West Shewa	35	23	65.7	17	48.6	31	88.6	25	71.4^b^	4/6	66.7	13/29	44.8^a^
Total	**200**	113	56.5	101	50.5	188	94	132	66	34/46	73.9^x^	80/152	(53)^y^
*P*-value (Zone)		0.14		0.97		0.10		**0.03**		0.60		**0.002**	

Abbreviations: aMPV, avian metapneumovirus; IBV, infectious bronchitis virus; ILTV, infectious laryngotracheitis virus; Mg, *Mycoplasma gallisepticum*; and NDV, Newcastle disease virus.

ab Means within columns not sharing a common letter in the superscript differ significantly (*P* < 0.05). *P* < 0.05 is boldfaced.

xy Significant difference between NDV vaccinated and unvaccinated chickens (*P* < 0.05). Statistically significant values are bolded.

**Table 4:** Individual chicken level seroprevalence of aMPV, ILTV, IBV, Mg, and NDV in two production systems.

Type of production	n	aMPV positive	ILTV positive	IBV positive	Mg positive	NDV positive (vaccinated)	NDV positive (non-vaccinated)
		N (%)	N (%)	N (%)	N (%)	N (%)	N (%)
Intensive	194	47 (24.2)^a^	19 (9.8)^a^	181 (93.3)	89 (45.9)	117/151 (78)^b^^x^	22/43 (51)^y^
Scavenging	301	119 (39.5)^b^	114 (37.9)^b^	267 (88.7)	144 (47.8)	17/33 (51.5)^a^	99/268 (37)
Total	495	166 (33.5)	133 (26.9)	448 (90.5)	233 (47.1)	134/184 (72.8)^x^	121/311 (39)^y^
*P*-value		**0.0004**	**<0.001**	0.09	0.67	**< 0.0001**	0.08

Abbreviations: aMPV, avian metapneumovirus; IBV, infectious bronchitis virus; ILTV, infectious laryngotracheitis virus; Mg, *Mycoplasma gallisepticum*; and NDV, Newcastle disease virus.

ab Means within columns not sharing a common letter in the superscript differ significantly (*P* < 0.05).

xy Significant difference between NDV vaccinated and unvaccinated chickens (*P* < 0.05). Statistically significant values are bolded.

#### Seroprevalence of the Pathogens in Different Zones

The seroprevalence of Mg and NDV at the holding level differed significantly between zones with a lower seroprevalence of Mg in East Shewa and significantly higher seroprevalence for NDV among unvaccinated chickens in the North Shewa zone (Table 3).

#### Seroprevalence of the Pathogens in Two Production Systems

The seroprevalence of ILTV at chicken (Table 4) and holding (Table 5) levels was significantly higher in the scavenging than in the intensive system. The same was true of aMPV but only at the individual chicken level (Table 4). The seroprevalence of Mg and IBV at individual and holding levels did not differ between production systems. The seroprevalence of NDV in vaccinated chickens was significantly higher in the intensive production system than the scavenging production system.

**Table 5:** Holding level seroprevalence of aMPV, ILTV, IBV, Mg, and NDV in two production systems.

Type of production	(N)	aMPV positive	ILTV positive	IBV positive	Mg positive	NDV positive (vaccinated)	NDV positive (non-vaccinated)
		N (%)	N (%)	N (%)	N (%)	N (%)	N (%)
Intensive (%)	**43**	25 (58.1)	13 (30.2)^b^	40 (93.0)	24 (55.8)	22/28 (78.6)	10/15 (66.7)
Scavenging (%)	**157**	88 (56.0)	88 (56.0)^a^	148 (94.3)	108 (68.8)	13/19 (66.7)	70/138 (50.7)
Total	**200**	113 (56.5)	101 (50.5)	188 (94)	132 (66)	34 (73.9)^x^	80 (53)^y^
*P* value		0.80	**0.0027**	0.76	0.11	0.37	0.25

Abbreviations: aMPV, avian metapneumovirus; IBV, infectious bronchitis virus; ILTV, infectious laryngotracheitis virus; Mg, *Mycoplasma gallisepticum*; and NDV, Newcastle disease virus.

ab Means within columns not sharing a common letter in the superscript differ significantly (*P* < 0.05).

xy Significant difference between NDV vaccinated and unvaccinated chickens (*P* < 0.05). Statistically significant values are bolded.

### Seroprevalence of the Pathogens in Different Breeds of Chickens

The seroprevalence of holdings by breed was calculated only for the scavenging production system since the intensive production system raised only exotic breeds. The seroprevalence of ILTV was significantly (*P* = 0.0001) higher in local and crossbred chickens than in exotic commercial layer chickens, but the reverse was the case for seroprevalence of NDV in unvaccinated chickens (Table 6).

**Table 6:** Seroprevalence of aMPV, ILTV, IBV, Mg, and NDV in different breed groups within the scavenging production system.

Breed category	(N)	aMPV positive	ILTV positive	IBV positive	Mg positive	NDV positive (vaccinated)	NDV positive (non-vaccinated)
		N (%)	N (%)	N (%)	N (%)	N (%)	N (%)
Exotic (%)	**144**	51 (35.4)	40 (27.8)^a^	128 (88.9)	78 (54.2)^b^	7/18 (38.9)	65/126 (51.6)^b^
Local & Crossbred (%)	**157**	68 (43.3)	74 (47.14)^b^	139 (88.5)	66 (42)^a^	10/15(66.7)^x^	34/142 (23.9)^a^^y^
Total	**301**	199 (39.5)	114 (37.9)	267 (88.7)	144 (47.8)	17/33 (51,5)^x^	99/268 (36.9)^y^
*P* value		0.16	**0.0005**	0.92	**0.035**	0.11	**<0.0001**

Abbreviations: aMPV, avian metapneumovirus; IBV, infectious bronchitis virus; ILTV, infectious laryngotracheitis virus; Mg, *Mycoplasma gallisepticum*; and NDV, Newcastle disease virus.

ab Means within columns not sharing a common letter in the superscript differ significantly (*P* < 0.05).

xy Significant difference between NDV vaccinated and unvaccinated chickens (*P* < 0.05). Statistically significant values are bolded.

### Odds Ratios for Seropositivity for the Different Pathogens

Seropositivity at the household level for a pathogen other than NDV tended to increase the risk of seropositivity for other pathogens with significant odds ratios above 4 for aMPV, IBV, and ILTV (Table 7). Similarly, there was a highly significant positive association between seropositivity to ILTV and Mg across households.

**Table 7:** The odds ratio of a seropositive result for a pathogen on being seropositive for the other pathogens at the holding level.

If also Positive to	Risk (Odds Ratio) of being positive to									
	ILTV		IBV		Mg		NDV vaccinated		NDV not vaccinated	
	OR	*P*=	OR	*P*=	OR	*P*=	OR	*P*=	OR	*P*=
AMPV	4.63	**<0.001**	7.21	**<0.004**	3.44	**<0.001**	0.7	0.73	1.0	0.96
ILTV			0.31	0.07	3.44	**<0.001**	1.9	0.45	0.6	0.13
IBV					2.03	0.2	8.4	**0.04**	6.7	0.08
MG							0.6	0.9	0.9	0.85

Significant *P* values are bolded.

Abbreviations: aMPV, avian metapneumovirus; IBV, infectious bronchitis virus; ILTV, infectious laryngotracheitis virus; Mg, *Mycoplasma gallisepticum*; and NDV, Newcastle disease virus.

### Frequency of Seropositivity to Multiple Pathogens in Unvaccinated Chickens

**Figure 2:**
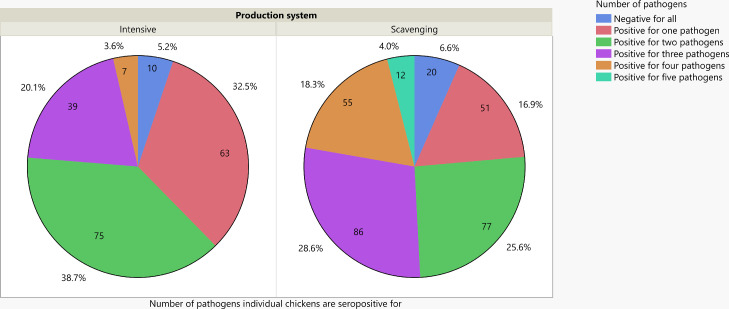
Proportion of chickens seropositive for 0, 1, 2, 3, 4 or 5 pathogens investigated in this study (aMPV, ILTV, IBV, Mg, and NDV). NVD is treated as a pathogen only in samples from non-NDV vaccinated chickens. Abbreviations: aMPV, avian metapneumovirus; IBV, infectious bronchitis virus; ILTV, infectious laryngotracheitis virus; Mg, *Mycoplasma gallisepticum*; and NDV, Newcastle disease virus.

**Figure 3:**
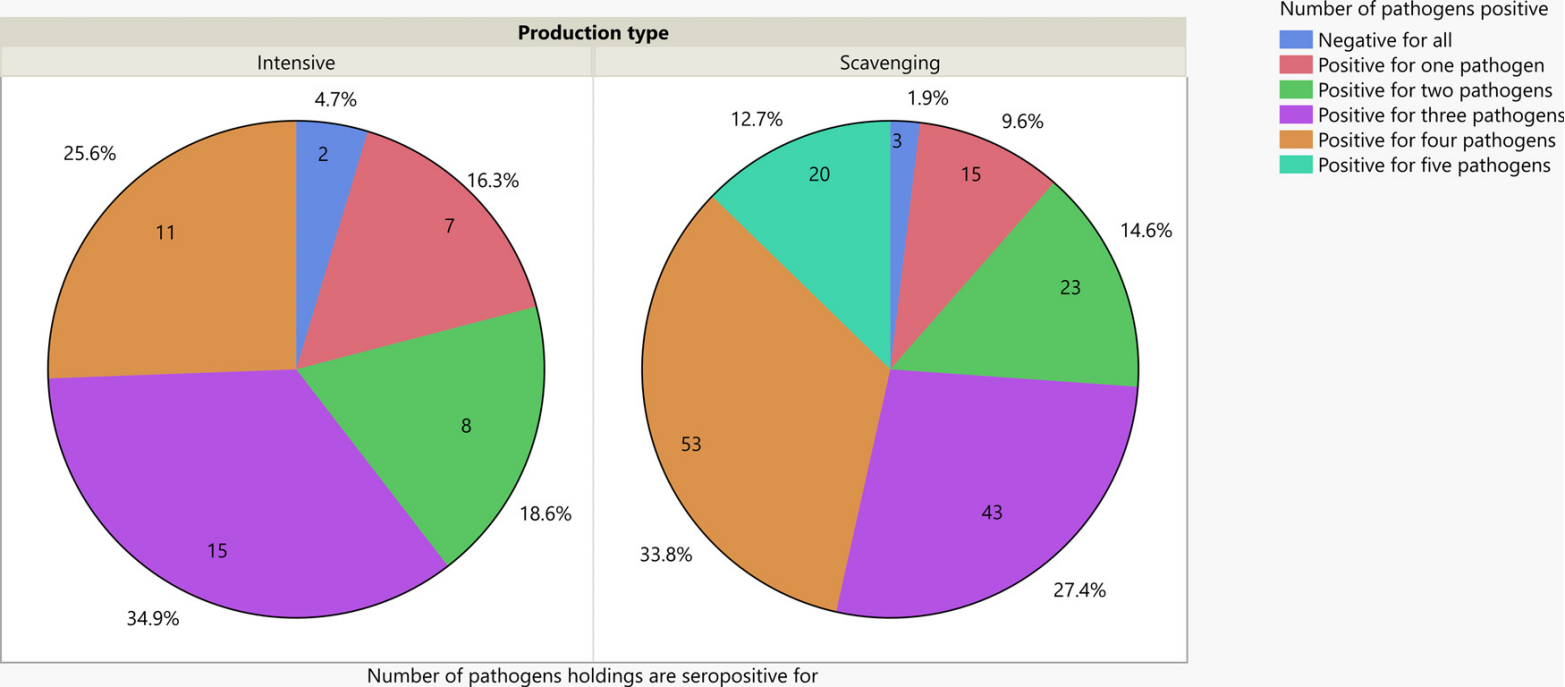
Proportion of holdings seropositive for 0, 1, 2, 3, 4, or 5 of the five pathogens investigated in this study (aMPV, ILTV, IBV, Mg, and NDV). NVD is treated as a pathogen only in samples from non-NDV vaccinated holdings. Abbreviations: aMPV, avian metapneumovirus; IBV, infectious bronchitis virus; ILTV, infectious laryngotracheitis virus; Mg, *Mycoplasma gallisepticum*; and NDV, Newcastle disease virus.

In this analysis, seropositivity to NDV was considered to represent infection by wild-type NDV if the chicken or holding was not vaccinated against NDV. The vast majority of chickens in both production systems were seropositive for at least one of the pathogens assessed (281/301 or 93.4% in the scavenging system and 184/194 or 94.8% in the intensive production system (Figure 2). However, there was a highly significant difference between the production systems in the proportions of chickens seropositive for 0, 1, 2, 3, 4, or 5 pathogens (*P* < 0.0001) with a higher mean number in the scavenging system (2.47) than the intensive system (1.85). The majority (153/301, 50.8%) of chickens in the scavenging production system was seropositive for 3 or more of the 5 pathogens tested, but the equivalent proportion in the intensive system was 46/194 (23.7%). Of 495 birds sampled, 12 were seropositive for all 5 pathogens, and these birds were all in scavenging production. At a holding level, the proportion of holdings seropositive for 3 or more pathogens in the intensive and scavenging production systems were 26/43 (60%) and 116/157 (74%), respectively (Figure 3). Thus, prior exposure to multiple pathogens was common, particularly in the scavenging system.

## DISCUSSION

This study aimed to ascertain the prevalence of respiratory pathogens in poultry-producing zones around the Ethiopian capital and their possible contribution to the fengil syndrome. There was moderate to high seroprevalence of all tested pathogens, including the first description of seroprevalence against aMPV in Ethiopia. There was evidence of a high level of exposure to multiple pathogens with seropositivity to aMPV, ILTV, IBV and Mg tending to be associated with exposure to other pathogens. The scavenging system had a higher level of exposure to multiple pathogens and possibly lower effectiveness of NDV vaccination given the lower NDV seropositive rate in NDV-vaccinated chickens in the scavenging than the intensive system. These findings in light of the original propositions are discussed in more detail below.

The first proposition that serological evidence of infection with aMPV, ILTV, IB, Mg, and NDV will be found to be widespread in the surveyed region was supported by the findings of the study. Holding level exposure to these pathogens at moderate to high prevalence levels (37–98%) was evident in all zones and production systems. The results suggest that these pathogens are endemic in the study area, with the wide range of antibody titers observed for all the pathogens most likely indicating different levels of infection or time since exposure to the pathogens. It is also the first report of widespread exposure to aMPV in the field, following initial detection in imported breeds at a government facility (Hutton et al., 2017). The seroprevalence of IBV (94%) in the current study is similar to 97% seroprevalence in central Ethiopia reported by (Shiferaw et al., 2022a) but, the individual chicken seroprevalence of Mg in the scavenging production system (48%) is lower than the 76% seroprevalence reported by (Shiferaw et al., 2022b). ILTV was reported for the first time in the West and North Shewa zones of Ethiopia. The widespread serological evidence of the circulation of these pathogens in the studied areas suggests their possible contribution to the fengil syndrome. This syndrome is ascribed to NDV but interestingly, among unvaccinated chickens in the scavenging system, the seropositivity rate for NDV was numerically lower than for any other pathogen tested. The characteristic clinical signs of the fengil syndrome, such as sneezing, rales, gasping, nasal discharge, and swelling of the sinuses could be caused by a combination of any of the 5 pathogens investigated in this study, or indeed several additional pathogens not investigated in this study.

The second proposition that the seroprevalence of the tested pathogens will differ between production systems and breeds was supported for some of the pathogens tested. The observed higher ILTV and aMPV seropositivity in the scavenging than intensive production system may relate to differences in husbandry with intensive production systems better able to restrict the introduction of pathogens to the flock. The reported seroprevalence of ILTV in this study in the intensive (30%) and scavenging (56%) production systems were higher than the 12% reported for large-scale commercial chicken farms and 34% in scavenging systems in Ethiopia by Tesfaye et al. (2019) in East Shewa and South Ethiopia. The reported lower seroprevalence for ILTV in large intensive farms could be related to better biosecurity implementation in large-scale systems compared to the small-scale intensive production systems in the present study. The reported seroprevalence of Mg (46%) in intensive production system is lower than the report by (Shiferaw et al., 2022b) who reported 70.6% seroprevalence in Bishoftu and Hawassa towns of Ethiopia. Overall, chickens in the scavenging production system were seropositive for a greater number of respiratory pathogens than those in the intensive system. This is not surprising as intensive production systems are characterized by chickens confined within their flocks with less opportunity for mixing with chickens from other flocks or wild birds. This highlights the importance of biosecurity arrangements in limiting exposure to respiratory pathogens.

NDV is endemic in Ethiopia and a known cause of high morbidity and mortality in intensive and scavenging production systems. In the present study, there was a non-significant trend (*P* = 0.08) for a higher seroprevalence of NDV among non-vaccinated chickens in the intensive system, than in the scavenging system. The reasons for such a trend are not known. The overall seroprevalence of NDV in unvaccinated scavenging chickens (38%) was higher than previously reported in other areas of the country (11% in Central Ethiopia and 26% in Northern Ethiopia; Derbew et al., 2016; Sori et al., 2016), although it was similar to the 32% reported by Tadesse et al. (2005) in central Ethiopia.

Breed effects on seroprevalence were observed for ILTV and NDV unvaccinated birds. The higher seropositivity of exotic chickens for NDV compared to local or crossbreed among non-vaccinated scavenging chickens may indicate greater susceptibility to the former. However, this should be interpreted with caution as it may also be due to the possible vaccination of exotic chickens at hatching and brooding facilities, with the owners being unaware of this, or better immune response to vaccination or exposure in exotic breeds. In contrast, seropositivity for ILTV was higher in local or crossbreed chickens, which could be because local breeds have a better survival rate following infection with ILTV. Alternatively, local breeds could be more susceptible to ILTV with a low mortality rate (Psifidi et al., (2014).

The third proposition that serological evidence of exposure to multiple agents will be common and exposure to ILTV, IBV, NDV, Mg, or aMPV will predispose to coinfection with other respiratory pathogens assessed was strongly supported by the findings of the current study. In the scavenging system, more than 50% of chickens and 74% of holdings were seropositive for 3 or more pathogens, and 6.6% of chickens and 12.7% of holdings were seropositive for all 5 pathogens tested. Overall, 60.5% of holdings were seropositive for 3 or more pathogens tested in the intensive systems. As this analysis excluded seropositive chickens and holdings due to ND vaccination and there was no history of vaccination for aMPV, ILTV, IBV, and Mg, the seropositivity suggests exposure to these pathogens. The immunosuppression induced by aMPV is known to predispose chickens to infection with other pathogens such as NDV, *Bordetella, Ornithobacterium*, and *E. coli* (Turpin et al., 2002; Jirjis et al., 2004; Marien et al., 2005; Psifidi et al., 2014; Bao et al., 2020; Swayne, 2020), which could be occurring in Ethiopia. It was also observed seropositivity to ILTV increased the odds of Mg seropositive results and vice versa. Co-infection with respiratory pathogens is commonly reported (Couto et al., 2016; Swayne, 2020) and is likely indicative of similar epidemiological factors involved in the transmission of these pathogens. However this should be properly assessed. The high level of exposure to multiple agents is supportive of the notion that fengil may represent a clinical syndrome with multiple causative agents rather than a specific disease. Only 2.5% of holdings overall were seronegative for all 5 pathogens. It highlights the challenges in breaking cycles of transmission by improved biosecurity measures, particularly in scavenging systems.

The fourth proposition that the seroprevalence of NDV infection will be higher in vaccinated than unvaccinated flocks was unsurprisingly supported by the findings. Vaccination practice for NDV is not common in the scavenging production system apart from some annual government-implemented campaigns in pilot areas. During the campaigns, farmers are expected to bring their chickens to a nominated vaccination area, sometimes involving travel over considerable distances. The stress to chickens associated with this process may reduce vaccination effectiveness. This, together with other factors associated with the implementation of vaccination in the field, may explain the significantly lower seropositivity rate amongst vaccinated chickens in the scavenging than in the intensive system. Poor vaccination results in the scavenging production system have also been reported by Mebrahtu et al. (2018). In Ethiopia, vaccination in the intensive production system is generally performed by private veterinarians, and periodic booster vaccinations are common. Despite significant production system differences in NDV seropositivity in vaccinated chickens at the individual chicken level, at the holding level NDV seropositivity in vaccinated groups was more similar between the 2 groups and not statistically significant.

## CONCLUSIONS

The study has revealed moderate to high seroprevalence of aMPV, ILTV, IBV, Mg, and NDV in the chickens in central Ethiopia, indicative of an endemic status of these pathogens. It is the first evidence of the widespread presence of aMPV in the field. Only 2.5% of the flocks investigated were serologically negative for the pathogens assessed. A high level of seropositivity to multiple agents was found, potentially implicating multiple pathogens in the causation of fengil in Ethiopia. Seropositivity for one pathogen tended to predispose to seropositivity for other pathogens, with aMPV, ILTV, IBV and Mg having the strongest associations. The seroprevalence of aMPV and ILTV was higher in chickens under the scavenging than in the intensive system, highlighting the likely role of containment and biosecurity in controlling these pathogens. Among ND vaccinated chickens, a higher rate of seropositivity was observed in the intensive than the scavenging system, suggestive of more effective vaccination practices in the former. This study highlights a potentially high respiratory disease burden in Ethiopian poultry production with potentially complex causation. Further studies are needed to evaluate the clinical significance of the pathogens assessed, risk factors for infection and disease, the pathogen strains involved and the economic impact of these diseases in Ethiopia. Such studies will inform the need for and design of appropriate control measures.

## DISCLOSURES

The authors declare that they have no conflict of interest.

## References

[bib0001] Alemu D., Degefe T., Ferede S., Nzietchueng S. & Roy D. (2008) Overview and background paper on Ethiopia’s poultry sector: Relevance for HPAI research in Ethiopia. *HPAI Africa/Indonesia Team Working Paper*.

[bib0002] Ali A. (2014). Surveillance of avian influenza virus in poultry and wild birds in Ethiopian Rift Valley Lakes. Int J Infect Dis.

[bib0003] Asfaw Y.T., Ameni G., Medhin G., Gumi B., Wieland B. (2021). Poultry health services in Ethiopia: availability of diagnostic, clinical, and vaccination services. Poult. Sci..

[bib0004] Bao Y., Yu M., Liu P., Hou F., Muhammad F., Wang Z., Li X., Zhang Z., Wang S., Chen Y. (2020). Novel inactivated subtype B Avian metapneumovirus vaccine induced humoral and cellular immune responses. Vaccines.

[bib0005] Batista I., Hoepers P., Silva M., Nunes P., Diniz D., Freitas A., Cossi M., Fonseca B. (2020). Circulation of major respiratory pathogens in backyard poultry and their association with clinical disease and biosecurity. Braz. J. Poult. Sci..

[bib0006] Bhuiyan Z.A., Ali M.Z., Moula M.M., Bary M.A., Arefin N., Giasuddin M., Khan Z.U.M. (2019). Seroprevalence of major avian respiratory diseases in broiler and sonali chicken in selected areas of Bangladesh. J. Adv. Vet. Anim. Res..

[bib0007] Birhan M., Temesgen M., Shite A., Berhane N., Bitew M., Gelaye E., Abayneh T., Getachew B. (2021). Seroprevalence and associated risk factors of infectious bronchitis virus in chicken in Northwest Ethiopia. Sci. World J..

[bib0008] Brown Jordan A., Gongora V., Hartley D., Oura C. (2018). A review of eight high-priority, economically important viral pathogens of poultry within the Caribbean region. Vet. Sci..

[bib0009] Chaka H., Goutard F., Bisschop S., Thompson P.N. (2012). Seroprevalence of Newcastle disease and other infectious diseases in backyard chickens at markets in eastern Shewa zone, Ethiopia. Poult. Sci..

[bib0010] Chaka H., Goutard F., Gil P., Abolnik C., Servan de Almeida R., Bisschop S., Thompson P.N. (2013). Serological and molecular investigation of Newcastle disease in household chicken flocks and associated markets in Eastern Shewa zone, Ethiopia. Trop. Anim. Health Prod..

[bib0011] Couto R.M., Braga J.F.V., Gomes S.Y., Resende M., Martins N.R., Ecco R. (2016). Natural concurrent infections associated with infectious laryngotracheitis in layer chickens. J. Appl. Poult. Res..

[bib0012] CSA (2021) Federal Democratic Republic of Ethiopia central statistical agency population projection of Ethiopia for all regions at Wereda level from 2020/2021. Addis Ababa, Ethiopia: Central Statistical Agency.

[bib0013] Derbew G., Getachew B., Haftu B. (2016). Sero-prevalence of Newcastle disease and its associated risk factors in village chickens at Alamata district, Southern Tigray, Ethiopia. Int. J. Eng. Dev. Res..

[bib0014] Dessie T. (1997) The role of scavenging poultry in integrated farming systems in Ethiopia. Livestock Feed Resources within Integrated Farming Systems. pp 377-398.

[bib0015] Dessie T., Ogle B. (2001). Village poultry production systems in the central highlands of Ethiopia. Trop. Anim. Health Prod..

[bib0016] Etsegenet Abera (2021). Ministry of Agriculture. Personal Communication.

[bib0017] Gharaibeh S., Algharaibeh G. (2007). Serological and molecular detection of avian pneumovirus in chickens with respiratory disease in Jordan. Poult. Sci..

[bib0018] Habte M., Debele S., Admassu B., Yinnessu A. (2015). Village chicken production performances assessment under scavenging management system in Amaro district, SNNPRS of Ethiopia. Wudpecker J*. Agric. Res*.

[bib0019] Habte T., Amare A., Bettridge J., Collins M., Christley R., Wigley P. (2017).

[bib0020] Hafez H.M. (2002). Diagnosis of Ornithobacterium rhinotracheale. In*t. J. Poult. S*ci..

[bib0021] Haji-Abdolvahab H., Ghalyanchilangeroudi A., Bahonar A., Ghafouri S.A., Marandi M.V., Mehrabadi M.H.F., Tehrani F. (2019). Prevalence of avian influenza, Newcastle disease, and infectious bronchitis viruses in broiler flocks infected with multifactorial respiratory diseases in Iran, 2015–2016. Trop. Anim. Health Prod..

[bib0022] Hutton S., Bettridge J., Christley R., Habte T., Ganapathy K. (2017). Detection of infectious bronchitis virus 793B, avian metapneumovirus, Mycoplasma gallisepticum and Mycoplasma synoviae in poultry in Ethiopia. Trop. Anim. Health Prod.

[bib0023] Jibril Y., Asfaw Y., Gebregziabher B., Issa A. (2018). Seroprevalence of Mycoplasma gallisepticum in domestic chickens, East Shewa, Ethiopia. Ethiopian Vet. J..

[bib0024] Jirjis F.F., Noll S.L., Halvorson D.A., Nagaraja K.V., Martin F., Shaw D.P. (2004). Effects of bacterial coinfection on the pathogenesis of avian pneumovirus infection in turkeys. Avian Dis..

[bib0025] Kryger K., Thomsen K., Whyte M., Dissing M. (2010).

[bib0026] Malik Y.S., Patnayak D.P., Goyal S.M. (2004). Detection of three avian respiratory viruses by single-tube multiplex reverse transcription–polymerase chain reaction assay. J. Vet. Diagn. Invest..

[bib0027] Mamo T., Yimer L. (2021). Serological investigation of Newcastle disease in selected districts of Buno Bedelle Zone, Ethiopia. Vet Med Res Rep.

[bib0028] Marien M., Decostere A., Martel A., Chiers K., Froyman R., Nauwynck H. (2005). Synergy between avian pneumovirus and Ornithobacterium rhinotracheale in turkeys. Avian Pathol..

[bib0029] Mazengia H. (2012). Review on major viral diseases of chickens reported in Ethiopia. J. Infect. Dis. Immun..

[bib0030] Mebrahtu K., Teshale S., Esatu W., Habte T., Gelaye E. (2018). Evaluation of spray and oral delivery of Newcastle disease I2 vaccine in chicken reared by smallholder farmers in central Ethiopia. BMC Vet. Res..

[bib0031] Nasser M., Lohr J., Mebratu G., Zessin K.-H., Baumann M., Ademe Z. (2000). Oral Newcastle disease vaccination trials in Ethiopia. Avian Pathol..

[bib0032] Psifidi A., Banos G., Matika O., Tadelle D., Christley R., Wigley P., Bettridge O., Hanotte T., Desta P.K. (2014). Proc10th World Congress Genet Appl Livestock Prod.

[bib0033] Roba Y.T., Tadesse D., Assefa Z., Tesfaye A. (2020). Seroprevalence of infectious laryngotracheitis disease in backyard chickens in villages of Ada'a district, Oromia, Ethiopia: first report. Trop. Anim. Health Prod..

[bib0034] Roussan D.A., Haddad R., Khawaldeh G. (2008). Molecular survey of avian respiratory pathogens in commercial broiler chicken flocks with respiratory diseases in Jordan. Poult. Sci..

[bib0035] Sambo E., Bettridge J., Dessie T., Amare A., Habte T., Wigley P., Christley R.M. (2015). Participatory evaluation of chicken health and production constraints in Ethiopia. Prev. Vet. Med..

[bib0036] Shankar B. (2008). Common respiratory diseases of poultry. Vet. World.

[bib0037] Shapiro B., Gebru G., Desta S., Negassa A., Nigussie K., Aboset G., Mechal H. (2015).

[bib0038] Shiferaw J., Dego T., Tefera M., Tamiru Y. (2022). Seroprevalence of Infectious bronchitis virus in broiler and layer farms of central Ethiopia. Biomed. Res. Int..

[bib0039] Shiferaw J., Shifara F., Tefera M., Feyisa A., Tamiru Y. (2022). Seroprevalence and associated risk factors of mycoplasma gallisepticum infection in poultry farms of Hawasa and Bishoftu, Central Ethiopia. Vet. Med..

[bib0040] Sori T., Eshetu A., Tesfaye A., Garoma A., Mengistu S. (2016). Seroprevalence of Newcastle disease in backyard chickens in Sebata Hawas District, central Ethiopia. World Ap*pl. Sci.* J..

[bib0041] Swayne D.E.B., Logue C.M., McDougald L.R., Nair V., Suarez D.L., Wit S., Grimes T., Johnson D., Kromm M., Prajitno T.Y., Rubinoff I., Zavala G., Martine (2020).

[bib0042] Tadesse S., Ashenafi H., Aschalew Z. (2005). Seroprevalence study of Newcastle disease in local chickens in central Ethiopia. Intl. J. Appl. Res. Vet. Med..

[bib0043] Tesfaye A., Sahle M., Sori T., Kassa T., Garoma A., Koran T., Dima C., Guyassa C., Hilu H., Guta S. (2019). Infectious laryngotracheitis virus in commercial and backyard chicken production systems in central and South Ethiopia (First report) ILT in Ethiopian poultry production. J. Appl. Poult. Res..

[bib0044] Turpin E.A., Perkins L.E., Swayne D.E. (2002). Experimental infection of turkeys with avian pneumovirus and either Newcastle disease virus or Escherichia coli. Avian Dis..

[bib0045] Zeleke A., Sori T., Gelaye E., Ayelet G. (2005). Newcastle disease in village chickens in the southern and rift valley districts in Ethiopia. Int. J. Poult. Sci..

